# Gut Microbiota Diversity of Preterm Neonates Is Associated With *Clostridioides Difficile* Colonization

**DOI:** 10.3389/fcimb.2022.907323

**Published:** 2022-07-06

**Authors:** Jeanne Couturier, Patricia Lepage, Sarah Jolivet, Johanne Delannoy, Victoria Mesa, Pierre-Yves Ancel, Jean-Christophe Rozé, Marie-José Butel, Frédéric Barbut, Julio Aires

**Affiliations:** ^1^ Université de Paris, Institut national de la santé et de la recherche médicale (INSERM) UMR S-1139 3PHM, Fédération hospitalo-universitaire (FHU) PREMA, F-75006, Paris, France; ^2^ National Reference Laboratory for Clostridioides difficile, Assistance Publique-Hôpitaux de Paris (AP-HP), Saint-Antoine Hospital, Paris, France; ^3^ Paris-Saclay University, institut national de recherche pour l'agriculture, l'alimentation et l'environnement (INRAE) AgroParisTech, Micalis Institute, Jouy-en-Josas, France; ^4^ Infection Control Unit, Assistance Publique-Hôpitaux de Paris (AP-HP), Saint-Antoine Hospital, Paris, France; ^5^ Université de Paris, Institut national de la santé et de la recherche médicale (INSERM) UMR 1153, Obstetrical, Perinatal and Pediatric Epidemiology Team (EPOPé), Center of Research in Epidemiology and Statistics (CRESS), Fédération hospitalo-universitaire (FHU) PREMA, Paris, France; ^6^ Unité de recherche clinique-Centre d'investigation clinique (URC-CIC) P1419, Hôpitaux universitaires Paris Centre (HUPC), Assistance Publique-Hôpitaux de Paris (AP-HP), Paris, France; ^7^ Pediatric Intensive Care Unit, Mothers’ and children’s Hospital, Nantes Teaching Hospital, Nantes, France

**Keywords:** *Clostridioides (Clostridium) difficile*, colonization, preterm neonates, gut microbiota, microbial diversity, 16S rRNA gene sequencing, necrotizing enterocolitis

## Abstract

In adults, *Clostridioides difficile* infections are associated with alterations of the intestinal bacterial populations. Although preterm neonates (PN) are frequently colonized by *C. difficile*, limited data are available regarding the relationship between *C. difficile* and the intestinal microbiota of this specific population. Therefore, we studied the intestinal microbiota of PN from two multicenter cohorts using high-throughput sequencing of the bacterial 16S rRNA gene. Our results showed that alpha diversity was significantly higher in children colonized by *C. difficile* than those without colonization. Beta diversity significantly differed between the groups. In multivariate analysis, *C. difficile* colonization was significantly associated with the absence of postnatal antibiotherapy and higher gestational age. Taxa belonging to the Lachnospiraceae, Enterobacteriaceae, Oscillospiraceae families and *Veillonella* sp. were positively associated with *C. difficile* colonization, whereas Bacteroidales and *Bifidobacterium breve* were negatively associated with *C. difficile* colonization. After adjustment for covariables, *Clostridioides, Rothia, Bifidobacterium, Veillonella, Eisenbergiella* genera and Enterobacterales were more abundant in the gut microbiota of colonized children. There was no significant association between *C. difficile* colonization and necrotizing enterocolitis in PN. Our results suggest that *C. difficile* colonization in PN is related to the establishment of physiological microbiota.

## Introduction

Asymptomatic *Clostridioides difficile* colonization with both toxigenic and non-toxigenic strains has been reported in 17–70% of healthy children less than three years of age (Enoch et al., 2011; Jangi and Lamont, 2010; Rousseau et al., 2011; Stoesser et al., 2017). Among pediatric populations, few studies have targeted *C. difficile* colonization in preterm neonates (PN); moreover, these studies are either outdated or based on culturing approaches (Cardines et al., 1988; el-Mohandes et al., 1993; Tina et al., 1994; Chang et al., 2012; Ferraris et al., 2012; Pichler et al., 2018). Depending on the study, the colonization rate in PN ranges from 0% (Tina et al., 1994) to 63% (Cardines et al., 1988) and increases during the first month of life. Preterm neonates are more exposed to hospital environments and antibiotics than full-term neonates. These factors influence gut colonization dynamics; for instance, the colonization rate is correlated to the hospital stay length and the resulting spore exposition (Bacon et al., 1988). Antibiotherapy is also associated with more frequent *C. difficile* colonization in PN (Tina et al., 1994; Pichler et al., 2018). Moreover, gut immaturity disturbs the gut microbiota, i.e., reduced bacterial diversity and richness and a low proportion of genera associated with health benefits (*Lactobacillus*, *Bacteroides*, *Bifidobacteria*) (Gewolb et al., 1999; Drell et al., 2014).

The link between intestinal dysbiosis and *C. difficile* infection (CDI) or colonization is gaining interest (Chen et al., 2019; Crobach et al., 2020; Han et al., 2020). *C. difficile* establishment in the adult gut occurs during dysbiosis, and CDI is associated with gut microbiota disruption leading to an increased risk of relapse (Barbut and Couturier, 2019). By sequencing the 16S rRNA gene of fecal samples from infected children aged 28–48 months, Ling *et al.* observed a decreased bacterial diversity compared to healthy controls (Ling et al., 2014). In addition, pediatric populations can act as reservoirs for adult CDI (Rousseau et al., 2012). Given the rising incidence of CDI in adults and children, understanding the early *C. difficile* colonization process and its role in gut microbiota establishment is of utmost importance.

The aim of this study was to evaluate *C. difficile* colonization in PN and its relationship with the intestinal microbiota. Using next-generation sequencing (NGS) of the 16S rRNA gene, we analyzed the intestinal microbiota of 599 PN from two multicenter cohorts, ClosNEC (n=116) and EPIFLORE (n=483), to assess bacterial signatures associated with *C. difficile* colonization. The impact of perinatal factors on colonization was also determined.

## Materials and Methods

### Subjects and Samples Collection

The fecal samples used in this study were obtained from PN recruited during two prospective multicenter studies, ClosNEC (2015–2016) and EPIFLORE (2011), including 20 neonatal intensive care unit facilities (NICUs). The aim of the ClosNEC cohort was to assess the relationship between bacterial colonization and necrotizing enterocolitis (NEC) in 159 recruited PN (<32 weeks of gestation) (Schönherr-Hellec et al., 2018). In this study, 118 fecal samples (35 from PN with NEC at its onset and 83 from matched controls) were analyzed using NGS 16S rRNA gene sequencing. The EPIFLORE cohort is an ancillary study of EPIPAGE 2, which aimed to describe the gut microbiota of PN born between 24–31 weeks of gestation (Rozé et al., 2017). A total of 751 children were recruited, and stool samples were collected in the first week [between day 3 (D3) and D7] (n=673) and 1 month [between D21 and D28 (n=620)]. The gut microbiota was analyzed by NGS for 142 and 486 fecal samples at one week (EPIFLORE_D7 dataset) and one month (EPIFLORE_D28 dataset), respectively. For 116 children whose samples were collected during the first week of life, a second sample was obtained at one month (EPIFLORE_D7_2 dataset). The flowcharts in **Figure S1** represent subject recruitment and stool samplings for both cohorts.

For the ClosNEC cohort (Clinical trial no. NCT02444624), approval was obtained from the National Data Protection Authority (Commission Nationale de l’Informatique et des Libertés, approval no. 915094) and the Consultative Committee on the Treatment of Information on Personal Health Data for Research Purposes (approval no. 15.055).

For the EPIFLORE cohort, approval was obtained from the National Data Protection Authority (Commission Nationale de l’Informatique et des Libertés, approval no. 911009), the Consultative Committee on the Treatment of Information on Personal Health Data for Research Purposes (approval no. 10.626), and the Committee for the Protection of People Participating in Biomedical Research (approval no. CPP SC-2873).

Informed consent was obtained from all the children’s parents involved in the study.

### Microbiota Analysis

Fresh fecal samples were collected from the diapers and stored at −80°C until microbiota analysis. Total DNA was extracted according to the International Human Microbiome Standards standard operating procedure 7 (IHMS_SOP 007) (Doré et al., 2015). The following template-specific primers were used: forward (5′-TACGGR(G/A)AGGCAGCAG-3′) and reverse (5′-TACCAGGGTATCTAAT-3′), targeting the V3-V4 hypervariable region of the 16S rRNA gene (Wilson et al., 1990). Quality control, sequencing library construction, and sequencing (MiSeq Illumina, San Diego, CA, United States) were performed using the Genotoul bioinformatics platform (Toulouse, France). Sequencing data were analyzed using the Galaxy-supported bioinformatics pipeline find, rapidly, OTUs with Galaxy solution (FROGS v3.0, http://bioinfo.genotoul.fr/, accessed 30/04/2020) (Afgan et al., 2018; Escudié et al., 2018). Primers were trimmed using Cutadapt (Martin, 2011). Sequences shorter than 410 bp and longer than 480 bp were excluded. Clustering was performed using the Swarm algorithm (Mahé et al., 2014) following the FROGS guidelines, that is, two successive steps with aggregation parameters d=1 and d=3. Chimeras were detected using the VSEARCH tool with the *de novo* UCHIME algorithm (Edgar et al., 2011; Rognes et al., 2016) and removed from further analysis. Filters were applied as follows: operational taxonomic units (OTUs) present in at least three samples and representing at least 0.00005% of the sequences were retained. Samples with fewer than 1000 amplified sequences were excluded from the analysis. OTUs were taxonomically classified using the basic local alignment search tool (BLAST) (Altschul et al., 1990) in the SILVA release 128 database (accessed 30/04/2020) (Quast et al., 2013). Taxonomic lineages were combined with a cluster abundance matrix to generate a raw OTU table.

Samples were defined as “*C. difficile* positive” (CD+) when at least 0.1% of the amplified sequences were assigned to the *C. difficile* OTU. The taxonomic affiliation of “*C. difficile*” was verified using BLAST (NCBI standard nucleotide blast https://blast.ncbi.nlm.nih.gov/Blast.cgi, accessed 30/04/2020) by comparing the OTU nucleotide sequence with the RefSeq_rna database. Taxonomic affiliation was considered “exact” if the identity percentage was 100%.

We compared the microbiota composition between the CD+ and CD− (“*C. difficile* negative”) groups using the FROGS Phyloseq v1.19.1 tool (accessed 02/05/2020) (McMurdie and Holmes, 2013). The data were normalized prior to diversity analysis. Results were considered significant at *p* < 0.05. Analysis of variance (ANOVA) was used to compare Chao 1 species richness estimation and Shannon diversity index between the patient groups. Microbial diversity was visualized using multidimensional scaling (MDS). To investigate associations between microbiota composition and *C. difficile* colonization, we performed a permutational multivariate analysis of variance (PERMANOVA) using the “Adonis” function with 9999 permutations, and Jaccard and weighted UniFrac (wUnifrac) distances. Biomarkers associated with colonization status were determined by the linear discriminant analysis (LDA) effect size (LefSe) algorithm (Segata et al., 2011).

Through a mixed linear model, the association of the most abundant OTUs transformed logarithmically with the patient individual variables were evaluated using the RStudio’s lme4 and nlme packages (Bates et al., 2015; Pinheiro et al., 2022). For OTUs that showed a significant association (*p < *0.05), the model was adjusted to control the effects of confounding variables. In statistical analysis, a *p < *0.05 value was considered significant.

Metagenomic functional prediction was carried out using Phylogenetic Investigation of Communities by Reconstruction of Unobserved States 2 (PICRUSt2 v2.5.0) based on 16S rRNA sequencing data (Langille et al., 2013; Douglas et al., 2020). The obtained prediction of metagenomic functional abundances was combined with descriptions from the KEGG Orthology (KO) database. Differentially present pathways between CD+ and CD− groups were analysed with welch test using STAMP (v2.1.3) (Parks et al., 2014).

### Isolation of *C. Difficile* by Culture and Strain Characterization

For *C. difficile* isolation, stool samples were processed as previously described (Couturier et al., 2020). Briefly, fecal samples were spread on the *C. difficile-*selective medium CLO-M (bioMérieux^®^, Marcy-l’Etoile, France) and incubated for 24 h at 37°C under anaerobic conditions (CO_2_:H_2_:N_2_, 10:10:80, anaerobic chamber). *C. difficile* isolates were identified using routine laboratory procedures. Toxigenic strains were identified by screening for *C. difficile tpi, tcdA*, and *tcdB* genes using multiplex PCR, as previously described (Lemee et al., 2004). Isolates were stored at −80°C in brain-heart infusion liquid medium supplemented with 15% glycerol.

### Perinatal Characteristics and Colonization Status

Clinical data, including birth mode, neonatal antibiotherapy, maternal antibiotherapy, and NEC onset, were prospectively collected during hospitalization until discharge for both PN cohorts. PN colonized by *C. difficile* were compared to those that were non-colonized. All variables were compared using the χ^2^ test or Fischer’s exact method, as required, or a non-parametric Mann-Whitney test for qualitative variables. Variables with *p* < 0.10 on univariable analysis were entered into a multivariate logistic regression model. A backward stepwise approach was used to identify independent predictors. Two-sided *p* < 0.05 defined significance. Statistical analyses were performed using Stata v15.1 software (StataCorp, College Station, Texas, USA).

## Results

### Microbiota Composition

The characteristics of the different datasets and results of the gut bacterial 16S rRNA gene sequencing analysis are listed in **Table 1**. Less than 1000 sequences were retrieved for two samples in the ClosNEC dataset and three samples in the EPIFLORE_D28 dataset (**Figure S1**). Therefore, these samples were excluded from the analysis. Rarefaction curves of the three datasets are available in **Supplementary material** (**Figures S2**–**S4**). Because of the low number of CD+ samples at D7 in the EPIFLORE_D7 dataset (n=2), we considered *C. difficile* colonization at D28 for the same child (n = 9) for the statistical analysis. Therefore, we used a subset of the EPIFLORE_D7 dataset called EPIFLORE_D7_2, which included children with samples at D7 and D28 (n=116). Groups were defined as *C. difficile*-positive at D28 (CD+D28) and *C. difficile-*negative at D28 (CD−D28), indicating the gut microbiota at D7 was compared according to the ulterior colonization status of the child at D28. For both children with a CD+ sample at D7, *C. difficile* was also detected in the D28 sample.

**Table 1:** Characteristics of datasets and results of the NGS analysis of the intestinal microbiota.

		DATASETS		
		ClosNEC (n = 116)	EPIFLORE_D28 (n = 483)	EPIFLORE_D7_2 (n = 116)
Birth term (weeks of gestation), Median, IQR		26, 25.5-28	29, 27-30	29, 27-30
Age at sampling time (days), Median, IQR		27, 17-36	23, 22-27	7, 6-9
N reads/sample, Median, min.-max.		30691, 7815–60122	23930, 1035–123937	25546.5, 1262–93173
N OTUs/sample, Median, min.-max.		42, 11-106	44, 9-154	41.5, 12-118
Percentage of main phyla, Median, IQR	Proteobacteria	28.41, 0.28-83.59	61.64, 1.32-87.91	44.03, 0.89-96.75
	Firmicutes	62.26,12.31-98.97	30.34, 9.13-94.88	18.18, 1.68-98.15
	Bacteroidota	0.02, 0.00-0.20	0.03, 0.01-0.13	0.09, 0.02-0.45
	Actinobacteriota	0.02, 0.01-0.13	0.02, 0.00-0.14	0.00, 0.00-0.03
N CD+ samples (%)		34 (29.3)	50 (10.4)	9*(7.8)

*Number of children with a CD+ sample at 1 month (CD+D28). IQR, interquartile range.

For all datasets, the microbiota was dominated by Firmicutes and Proteobacteria, with important inter-individual variations (**Table 1**). Among Proteobacteria, the most abundant family was Enterobacteriaceae, with median proportions of 10.63% [interquartile (IQR) 0.11–79.38], 57.33% (IQR 0.53–86.71), and 6.8% (IQR 0.51–95.95) for ClosNEC, EPIFLORE_D28, and EPIFLORE_D7_2, respectively. The most represented families of Firmicutes were Clostridiaceae for ClosNEC, Enterococcaceae for EPIFLORE_D28, and Staphylococcaceae for EPIFLORE_D7_2, with median proportions of 1.11% (IQR 0.03–43.8), 4.00% (IQR 0.28–17.28), and 2.07% (IQR 0.13–42.22), respectively. The median proportions of Lactobacillaceae and Bifidobacteriaceae were < 0.01% in all datasets.

Culture results were compared to *C. difficile* detection by NGS of the 16S rRNA gene for samples from the ClosNEC cohort (n=116) (**Table S1**). The overall agreement between the two methods was 86.2%. Among the five culture-positive NGS-negative samples, *C. difficile* sequences were detected in two samples, but in proportion < 0.1%, and therefore defined as “CD−”. Among the 28 C*. difficile* strains, 26 (92.9%) were non-toxigenic, and 2 (7.1%) were toxigenic. Owing to the low number of samples analyzed by both culture and NGS (n=27), we could not compare the results from both methods in the EPIFLORE cohort.

### Comparison of the Intestinal Microbiota Composition and Function According to *C. Difficile* Colonization

**Figure 1:**
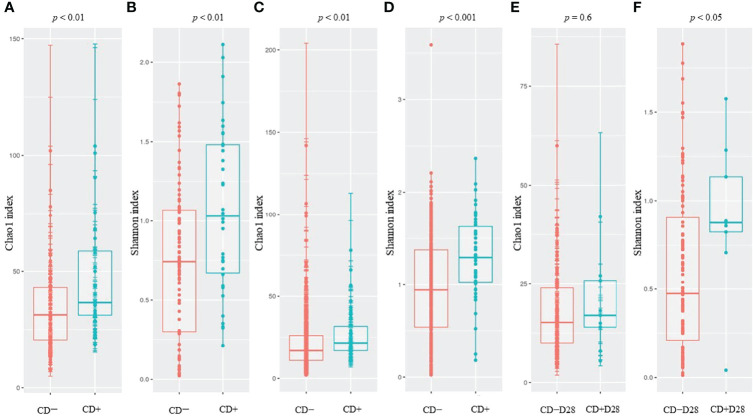
Comparison of the Chao1 and Shannon indices for ClosNEC [**(A)** and **(B)**], EPIFLORE_D28 [**(C)** and **(D)**] and EPIFLORE_D7_2 [**(E)** and **(F)**], respectively. Red, CD− (ClosNEC, EPIFLORE_D28) or CD−D28 (EPIFLORE_D7_2); blue, CD+ (ClosNEC, EPIFLORE_D28) or CD+D28 (EPIFLORE_D7_2). Boxplots represent the median and the 1^st^ and 3^rd^ quartiles.

We evaluated the differences in alpha diversity between CD+ and CD− samples (ClosNEC and EPIFLORE_D28 datasets) and CD+D28 and CD−D28 samples (EPIFLORE_D7_2 dataset). The median Chao1 and Shannon indices of CD+ samples were significantly higher than that of CD− samples (*p* < 0.01 and *p* < 0.01 for ClosNEC, respectively; *p* < 0.01 and *p* < 0.001 for EPIFLORE_D28, respectively) (**Figure 1**). At the first week of life, the median Shannon index in samples of PN colonized by *C. difficile* at one month (CD+D28 samples) was also higher than that of PN not colonized at one month (CD−D28 samples) (EPIFLORE_D7_2, *p* < 0.05) (**Figure 1**).

**Figure 2:**
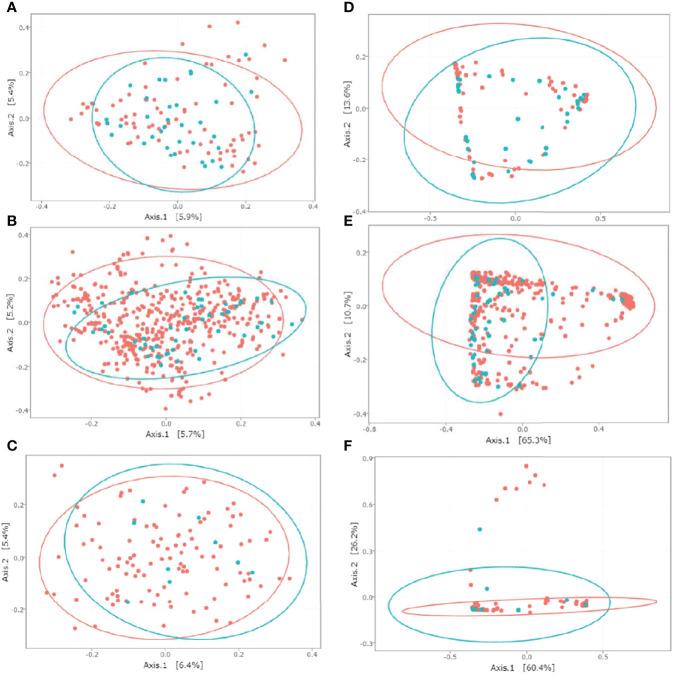
MDS representation of the intestinal microbiota based on Jaccard and wUnifrac indices for ClosNEC [**(A)** and **(D)**], EPIFLORE_D28 [**(B)** and **(E)**] and EPIFLORE_D7_2 [**(C)** and **(F)**], respectively. Each sample is represented by a color according to its category: red, CD− (ClosNEC, EPIFLORE_D28) or CD−D28 (EPIFLORE_D7_2); blue, CD+ (ClosNEC, EPIFLORE_D28) or CD+D28 (EPIFLORE_D7_2). The percentage of variation explained by the two first MDS dimensions is indicated on the respective axes.

To evaluate the beta diversity between CD-colonized and non-colonized groups, we performed MDS using Jaccard and wUnifrac matrices for the three datasets (**Figure 2**). Jaccard matrix analysis showed that the CD+ and CD− groups clustered separately for both the ClosNEC and EPIFLORE_D28 datasets (*p* < 0.001 and *p* < 0.001, PERMANOVA), as well as the CD+D28 and CD−D28 groups for the EPIFLORE_D7_2 dataset (*p* < 0.05, PERMANOVA). In the wUnifrac analysis, groups clustered separately only for the EPIFLORE_D28 dataset (*p* < 0.001 by PERMANOVA).

Functional predictions identified 22 and 76 differentially present KO pathways in ClosNEC (**Figure S5**) and EPIFLORE_D28 (**Figure S6**) datasets, respectively. In the ClosNEC dataset, the largest significant differences were pathways for energy metabolism, starch and sucrose metabolism, peptidases and amino acid metabolism; those pathways had higher proportions in the CD+ group. Energy metabolism, starch and sucrose metabolism, and peptidases had also higher proportions in the CD+ group of the EPIFLORE_D28 dataset. Largest significant differences in this dataset included bacterial motility proteins, flagellar assembly, and sporulation (higher in the CD+ group); DNA repair and recombination proteins, and ribosome (higher in the CD− group).

### Association of Perinatal Factors With the Colonization Status

Using univariate analysis, perinatal factors significantly associated with *C. difficile* colonization at one month in the EPIFLORE cohort included the absence of postnatal antibiotherapy [odds ratio (OR) 0.35, 95% confidence interval (CI) 0.17–0.72, *p* = 0.004], enterotype 2 profile as described by Rozé *et al.* (Rozé et al., 2020) (OR 2.36, 95% CI 1.18–4.72, *p* = 0.015), and a higher gestational age (OR 1.25, 95% CI 1.05–1.47, p = 0.010) (**Table 2**). In multivariate analysis, the only significantly associated variable was the absence of postnatal antibiotherapy. The most frequently used antibiotics in the EPIFLORE cohort were vancomycin (34%), aminosides (27%) and third generation cephalosporins (22%). Among PN who received a postnatal antibiotherapy, 93% were treated by at least two different antibiotics.

**Table 2:** Factors associated with *C. difficile* colonization at 1 month in the EPIFLORE cohort.

Variable	N neonates CD− (%)	N neonates CD+ (%)		Univariate analysis		Multivariate analysis	
				OR (95% CI)	*p*	Adjusted OR (95% CI)	*p*
Postnatal antibiotherapy after D3							
No	248 (58)	40 (80)		1.0			
Yes	177 (42)	10 (20)		0.35 (0.17.-0.72)	**0.004** *	0.35 (0.17-0.72)	**0.004**
Antibiotherapy at birth							
No	59 (16)	5 (11)		1.0			
Yes	300 (84)	40 (89)		1.57 (0.60-4.15)	0.36		
Transit during the first week							
Irregular	162 (40)	17 (37)		1.0			
Regular	243 (60)	29 (63)		1.14 (0.61-2.14)	0.69		
Enterotypes as defined by Rozé *et al.* (Rozé et al., 2020)							
1,3,4 and 5	370 (87)	37 (74)		1.0			
2	55 (13)	13 (26)		2.36 (1.18-4.72)	**0.015** *		
Maternal antibiotherapy							
No	202 (48)	21 (42)		1.0			
Yes	223 (52)	29 (58)		1.25 (0.69-2.26)	0.46		
Birth mode							
Vaginal	143 (34)	23 (46)		1.0			
C-section	281 (66)	27 (54)		0.60 (0.33-1.09)	0.09		
Skin to skin							
No	149 (38)	14 (32)		1.0			
Yes	246 (62)	30 (68)		1.30 (0.67-2.53)	0.44		
NEC							
No	408 (97)	50 (100)		–			
Yes	13 (3)	0 (0)		–	–		
Gestational age, weeks of gestation (median, IQR)							
	29 (27-30)	30 (29-31)		1.25 (1.05-1.47)	**0.010** *		

Statistically significant values of p are in bold type. CI, confidence interval; IQR, interquartile range; OR, odds ratio; NEC, necrotizing enterocolitis. *Variables included in the multivariate analysis.

In univariate analysis, factors significantly associated with *C. difficile* colonization in the ClosNEC cohort were transit considered normal at D7 (OR 3.83, 95% CI 1.06–13.91, *p* = 0.041) and a higher gestational age (OR 1.26, 95% CI 1.03–1.52, *p* = 0.019) (**Table 3**). In the multivariate analysis, higher gestational age was the only significantly associated factor. PN of the ClosNEC cohort received third generation cephalosporins (68%), aminosides (59%), vancomycin (59%) or other antibiotics (30%). Among PN who received a postnatal antibiotherapy, 97% were treated by at least two different antibiotics. Maternal antibiotherapy, birth mode, and NEC were not identified as factors associated with *C. difficile* colonization.

**Table 3:** Factors associated with *C. difficile* colonization in the ClosNEC cohort.

Variable	N neonates CD− (%)	N neonates CD+ (%)	Univariate analysis		Multivariate analysis	
			OR (95% CI)	*p*	Adjusted OR (95% CI)	*p*
Neonatal antibiotherapy						
No	17 (21)	7 (21)	1.0			
Yes	65 (79)	27 (79)	1.01 (0.37-2.71)	0.99		
Maternal antibiotherapy						
No	42 (54)	19 (58)	1.0			
Yes	36 (46)	14 (42)	0.86 (0.38-1.95)	0.72		
Transit at D7 considered as normal						
No	23 (30)	3 (10)	1.0			
Yes	54 (70)	27 (90)	3.83 (1.06-13.91)	**0.041** *		
Birth mode						
Vaginal	36 (44)	11 (32)	1.0			
C-section	46 (56)	23 (68)	1.64 (0.71-3.79)	0.25		
NEC						
No	59 (72)	22 (65)	1.0			
Yes	23 (28)	12 (35)	1.40 (0.60-3.28)	0.44		
Gestational age, weeks of gestation (median, IQR)						
	28 (26-30)	29 (28-31)	1.26 (1.03-1.52)	**0.019** *	1.26 (1.03-1.52)	**0.019**

Statistically significant values of p are in bold type. CI, confidence interval; IQR, interquartile range; OR, odds ratio; NEC, necrotizing enterocolitis. * Variables included in the multivariate analysis.

### Bacterial Biomarkers Associated With the Colonization Status

**Figure 3:**
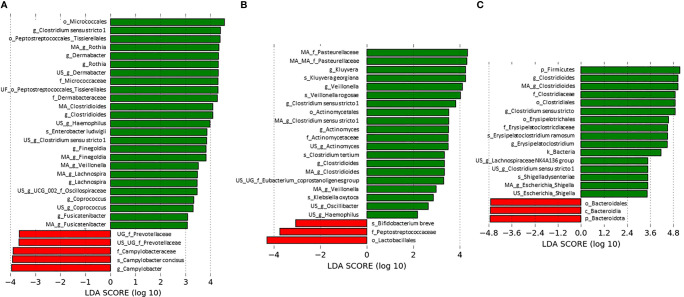
Most differentially abundant taxa associated with *C. difficile* absence (red) or presence (green) **(A)** at the sampling time for the ClosNEC dataset, **(B)** at the sampling time for the EPIFLORE_D28 dataset and **(C)** at D28 for the EPIFLORE_D7_2 dataset. LDA scores are expressed in log_10_ values. Only taxa meeting an LDA significant threshold > 2 are shown. MA: multi-affiliation; US: unknown species; UG: unknown genus; UF: unknown family; k: kingdom; p: phylum; c: class; o: order; f: family; g: genus; s: species.

Bacterial biomarkers associated with the presence or absence of *C. difficile* in each dataset (at the sampling time for ClosNEC and EPIFLORE_D28, and 1 month for EPIFLORE_D7_2) are shown in **Figure 3**. We identified several biomarkers associated with *C. difficile* colonization, particularly *Veillonella* sp., and taxa belonging to families such as Lachnospiraceae, Oscillospiraceae, and Enterobacteriaceae (*Enterobacter ludwigii* for ClosNEC*, Escherichia–Shigella* for EPIFLORE_D7_2, *Klebsiella oxytoca*, and *Kluyvera* for EPIFLORE_D28). *Bifidobacterium breve* and Bacteroidales were significantly more abundant in samples negative for *C. difficile*.

From the mixed linear model analysis, variables identified as significantly associated with the colonization status were integrated with the specifically associated OTUs (**Table S2**), i.e. for ClosNEC, gestational age and transit at D7 considered as normal, and for EPIFLORE, postnatal antibiotherapy after D3, enterotype profile according to Rozé *et al.* (Rozé et al., 2020), and gestational age. For OTUs that showed a significant association (*p* < 0.05), the model was fitted to control the effect of other variables. For ClosNEC, 4 OTUs maintained a positive significant interaction with the CD+ group after fitting the model: *Clostridioides, Rothia, Bifidobacterium* and *Veillonella.* For EPIFLORE, *Clostridioides*, Enterobacterales and *Eisenbergiella* maintained a positive significant interaction with the CD+ group after fitting the model.

## Discussion

We investigated the intestinal microbiota of PN, focusing on variations in bacterial populations associated with *C. difficile* colonization. This is an original study, as it included PN from two multicentric cohorts with microbiota analysis based on both culture analysis and 16S rRNA gene sequencing.

Our results showed that the median number of OTUs in each sample ranged from 41.5–44 for the three datasets analyzed, confirming the low richness of the intestinal ecosystem in PN (Drell et al., 2014). The most represented phyla were Proteobacteria and Firmicutes, whereas the abundance of Bifidobacteriaceae and Lactobacillaceae was low (median < 0.01%), in agreement with previous data (Drell et al., 2014; Ho et al., 2018). Among the PN of the EPIFLORE cohort with a first-week sample (n=142), only two (1.3%) were CD+, whereas the prevalence of *C. difficile* in stools sampled at one month was 10.4% (50/483). *C. difficile* was detected in 29.3% of the samples (n=116) for the ClosNEC cohort. These colonization rates were lower than those observed in the previous PREMAFLORA cohort (Ferraris et al., 2019) where 20% of PN were colonized at one week and 61% at one month. This difference could be explained by the fact that PREMAFLORA was a single-center study with potential cross-transmission of *C. difficile* within the ward. Moreover, PN included in PREMAFLORA had a higher gestational age than that in the ClosNEC and EPIFLORE cohorts (median gestational age 32 weeks, range 24–36 weeks). Notably, most strains isolated by culture were non-toxigenic (92.9%).

The reported rates of *C. difficile* colonization in PN are highly variable and originate primarily from outdated, single-center, culture-based studies (Cardines et al., 1988; Chang et al., 2012; Pichler et al., 2018). The *C. difficile* colonization rates depend on the detection method used. In our study, the agreement between bacterial culture and NGS of the 16S rRNA gene was 86.2%. The detection threshold for the culture-based method was 3.3 log_10_ CFU/g stools (Ferraris et al., 2019). The detection limits of NGS-based methods vary from one study to another, particularly because of the use of different primers targeting different regions of the 16S rRNA gene. In samples less complex than feces (urine, water), bacteria can be detected at 10^1^–10^2^ CFU/mL (Sabat et al., 2017; Brandt and Albertsen, 2018). However, it has been suggested that sequence-based techniques can miss bacteria present in the adult gut microbiota at concentrations lower than 10^5^ CFU/mL (Lagier et al., 2012). Detection of the 16S rRNA gene does not indicate the viability of the microorganisms in the sample. Crobach *et al.* assigned patients to colonized, infected, and control groups according to culture results, and sequences affiliated with the *Clostridioides* OTU were detected in only 26/41 (63.4%) colonized patients and 38/41 (92.7%) infected patients (Crobach et al., 2020).

We showed that alpha diversity at D28 (for both ClosNEC and EPIFLORE_D28 datasets) increased when *C. difficile* was detected in stool samples. Alpha diversity at D7 (EPIFLORE_D7_2 dataset) was also higher when *C. difficile* was detected in samples from the same PN at D28. These findings are unusual compared to available data from 16S rRNA gene NGS analysis suggesting that *C. difficile* colonization is associated with a decreased alpha diversity of the gut microbiota in adults and children (Chen et al., 2019; Han et al., 2019; Crobach et al., 2020; Han et al., 2020). In a study performed in two Dutch NICUs, Crobach *et al.* reported that the Shannon index was significantly higher in 43 control adult individuals than in 41 C*. difficile*-colonized or 41 -infected patients (*p* < 0.01), whereas no difference was seen between colonized and infected patients (Crobach et al., 2020). Similar results were obtained in a Korean single-center study including 99 adult patients, where the mean Chao1 index of patients who were *tcdB*-positive was significantly lower than that of the control group (*p* < 0.001) (Han et al., 2019). In a pediatric population (median age 14 years, range 4–19 years, n=113), alpha diversity was also significantly lower in the *C. difficile*-colonized group (*p* = 0.029) (Chen et al., 2019). However, another study showed no difference in alpha diversity (Chao1 index) between *C. difficile*-colonized (n=93) and control (n=93) adults (*p* = 0.797) (Han et al., 2020). Unlike older populations, our results suggest that in PN, *C. difficile* colonization is concomitant with physiological establishment and diversification of the gut microbiota. Moreover, PN included in our study were mostly colonized by non-toxigenic strains, which may have different effects on the gut microbiota.

PERMANOVA analysis of the gut microbiota in the ClosNEC cohort showed that beta diversity estimated by the Jaccard index differed significantly between CD+ and CD− samples, but not according to the wUnifrac method, suggesting that abundant and phylogenetically-related species were shared. In the EPIFLORE cohort, the microbiota composition significantly differed between CD+ and CD− samples at D28 (EPIFLORE_D28 dataset) for both beta diversity matrices. For samples at D7 (EPIFLORE_D7_2 dataset), only beta diversity according to the Jaccard index showed a significantly different composition between PN subsequently colonized at D28 and PN not colonized at D28. These results align with the progressive maturation and diversification of the gut microbiota between one week and one month of life.

The functional pathway prediction analysis showed that several KO pathways differed according to the colonization status. Of note, *C. difficile-*colonized PN had higher proportions of energy metabolism, starch and sucrose metabolism, and peptidases pathways in both ClosNEC and EPIFLORE cohorts. However, this analysis is only predictive and further studies are needed to confirm these findings.

Using LefSe analysis, we identified several bacterial biomarkers associated with *C. difficile* colonization of PN, including *Veillonella*, Lachnospiraceae, Oscillospiraceae, and Enterobacteriaceae. *Veillonella* are commensal bacteria of the oral cavity that have been previously associated with CDI (Han et al., 2020). *Veillonella* is associated with low rates of coprostanol, a cholesterol metabolite. Antharam *et al.* discussed these metabolic abilities as causes of CDI susceptibility, although the molecular mechanisms involved remain unknown (Antharam et al., 2016). Lachnospiraceae family have been previously associated with *C. difficile* colonization in children aged 4–21 (Chen et al., 2019). Bacteria belonging to the Lachnospiraceae family produce short-chain fatty acids, which are end products of dietary carbohydrates with a beneficial effect on host health (Wong et al., 2006). We hypothesized that the Lachnospiraceae taxa play a role in the permissiveness of the microbiota in *C. difficile* colonization. Our results on Enterobacteriaceae are consistent with those of previous studies on full-term neonates (Rousseau et al., 2011) and adult patients (Han et al., 2020). A high abundance of facultative anaerobic bacteria, fostering an anaerobic environment by consuming oxygen, has been reported to enable *C. difficile* colonization (Rousseau et al., 2011). Contradictory data are available for other biomarkers such as Oscillospiraceae. In a Chinese study, *Oscillibacter* was more abundant in healthy children than in infected children (Ling et al., 2014), whereas *Oscillospira* was more abundant in *C. difficile*-colonized than in infected adults (Han et al., 2019). We showed that *Bifidobacterium breve* and Bacteroidales, previously reported to protect against *C. difficile* colonization (Rousseau et al., 2011) and infection (Ling et al., 2014), were more abundant in PN without *C. difficile* colonization. The barrier effect of these taxa could explain our results (Roberfroid et al., 2010; Yang and Yang, 2019). To further identify these potential mechanisms, metabolite measures in stools and competition assays between *C. difficile* and identified biomarkers are necessary.

Using a mixed linear model, we identified several bacterial groups more abundant in the microbiota of CD+ children after adjustment for covariables, i.e. in the ClosNEC cohort, *Clostridioides, Rothia, Bifidobacterium* and *Veillonella*, and in the EPIFLORE cohort, *Clostridioides*, Enterobacterales and *Eisenbergiella.* With both LefSe analysis and the mixed linear model, bacterial biomarkers associated with the presence or absence of *C. difficile* were different between ClosNEC and EPIFLORE cohorts. This difference might be explained by i) the fact that the ClosNEC cohort included more children suffering from NEC than EPIFLORE cohort, ii) the fact that PN in the ClosNEC cohort received more frequently antibiotics, iii) the fact that the range of sampling times was different between the two cohorts (**Table 1**).

The *C. difficile* colonization rate increased when PN did not receive postnatal antibiotics (EPIFLORE) and had a higher gestational age (ClosNEC). Our results are unusual compared to those of other studies in adults showing that *C. difficile* is established in the gut as a result of dysbiosis, especially after antibiotherapy (Vincent et al., 2013). Several hypotheses may explain the relationship between antibiotics, the gut microbiota and *C. difficile* colonization: antibiotics might protect from colonization either *via* a direct activity on *C. difficile;* or *via* the induced dysbiosis which would be unfavourable for *C. difficile* implantation. In the EPIFLORE cohort, the fact that predictive differences in the gut microbiota diversity were observed at D7 between PN who would be colonized or not at D28 favours the latter hypothesis. The most frequently received antibiotics by PN of both cohorts were third generation cephalosporins, aminosides, and IV vancomycin, which are inefficient against *C. difficile.* Our statistical analyses corroborated the higher microbial diversity in colonized PN in accordance with the physiological nature of *C. difficile* implantation. Notably, we showed that *C. difficile* colonization did not differ between PN with or without NEC, supporting the fact that *C. difficile* is not involved in NEC pathogenesis (Lishman et al. 1984; Waligora-Dupriet et al., 2005).

This study has several limitations. When applying filters for OTUs, we lowered the minimum abundance threshold from 0.005% (recommended in (Bokulich et al., 2013)) to 0.00005% to account for the scarcity of PN microbiota (Drell et al., 2014) and to maintain potentially rare OTUs. To avoid artifactual OTUs resulting from sequencing errors, we retained OTUs present in at least three samples. Owing to highly variable sequencing coverage among samples, normalization before diversity analyses leads to a potential loss of rare OTUs in samples with a high number of amplified sequences. The small number of PN included in some groups, particularly children of the EPIFLORE cohort with a sample at D7 and subsequently colonized by *C. difficile* at D28 (n=9), reduced statistical power. This could partly explain why few diversity changes were observed between the groups in the EPIFLORE_D7_2 dataset. The variable “*C. difficile* presence or absence”-defining groups for microbiota analysis is based on the detection of sequences corresponding to *C. difficile* and not culture-based.

The strengths of this study are the high number of PN included (n total = 599), their recruitment from multicentric cohorts, and the extensive clinical data collected. Fecal samples obtained at both D7 and D28 for 116 PN enabled an analysis of the intestinal microbiota predictive of the subsequent colonization status, which is of utmost interest.

## Conclusions

This study provides new insights into understanding *C. difficile* colonization in PN and the associated gut microbiota modifications. Variations in bacterial populations and associated perinatal factors favor physiological *C. difficile* colonization, occurring in PN with a less disturbed microbiota. However, the timeline of this process and the mechanisms regulating the relationship between *C. difficile* and the establishment of gut microbiota are yet to be explained.

## Data Availability Statement

The datasets presented in this study can be found in online repositories. The names of the repository/repositories and accession number(s) can be found below: https://www.ncbi.nlm.nih.gov/, PRJNA819954.

## Ethics Statement

For the ClosNEC cohort (Clinical trial no. NCT02444624), approval was obtained from the National Data Protection Authority (Commission Nationale de l’Informatique et des Libertés, approval no. 915094) and the Consultative Committee on the Treatment of Information on Personal Health Data for Research Purposes (approval no. 15.055). For the EPIFLORE cohort, approval was obtained from the National Data Protection Authority (Commission Nationale de l’Informatique et des Libertés, approval no.911009), the Consultative Committee on the Treatment of Information on Personal Health Data for Research Purposes (approval no.10.626), and the Committee for the Protection of People Participating in Biomedical Research (approval no. CPP SC-2873). Written informed consent to participate in this study was provided by the participants’ legal guardian/next of kin.

## Author Contributions

Conceptualization, JA, M-JB, and FB. Methodology, JA, FB, and JC. Software, PL, VM, and JC. Validation, JA, FB, and JC. Formal analysis, JC, PL, JD, VM, and SJ. Investigation, JC and JD. Resources, JA, P-YA, M-JB, PL, and J-CR. Data curation, JC and JD. Writing—original draft preparation, JC. Writing—review and editing, all authors. Visualization, JC. Supervision, JA, and FB. Project administration, JA, FB, and JC. Funding acquisition, JA, P-YA, M-JB, and J-CR. All authors have read and agreed to the published version of the manuscript. All authors contributed to the article and approved the submitted version.

## Funding

This research was funded by the Agence Nationale de la Recherche (grants ANR-12-SV, ANR-12-BSV3-0025001/EPIFLORE, and ANR-13-PRTS-0018) and the PremUp Foundation.

## Conflict of Interest

The authors declare that the research was conducted in the absence of any commercial or financial relationships that could be construed as a potential conflict of interest.

## Publisher’s Note

All claims expressed in this article are solely those of the authors and do not necessarily represent those of their affiliated organizations, or those of the publisher, the editors and the reviewers. Any product that may be evaluated in this article, or claim that may be made by its manufacturer, is not guaranteed or endorsed by the publisher.
