# Supplementary material for: Gut Microbiota Diversity of Preterm Neonates Is Associated With Clostridioides Difficile Colonization
Source: Front Cell Infect Microbiol. 2022 Jul 6;12:907323. doi: 10.3389/fcimb.2022.907323 (PMC9296818; doi:10.3389/fcimb.2022.907323)
Supplement: Supplementary file 1 [file DataSheet_1.docx]

## Supplementary Figures

**
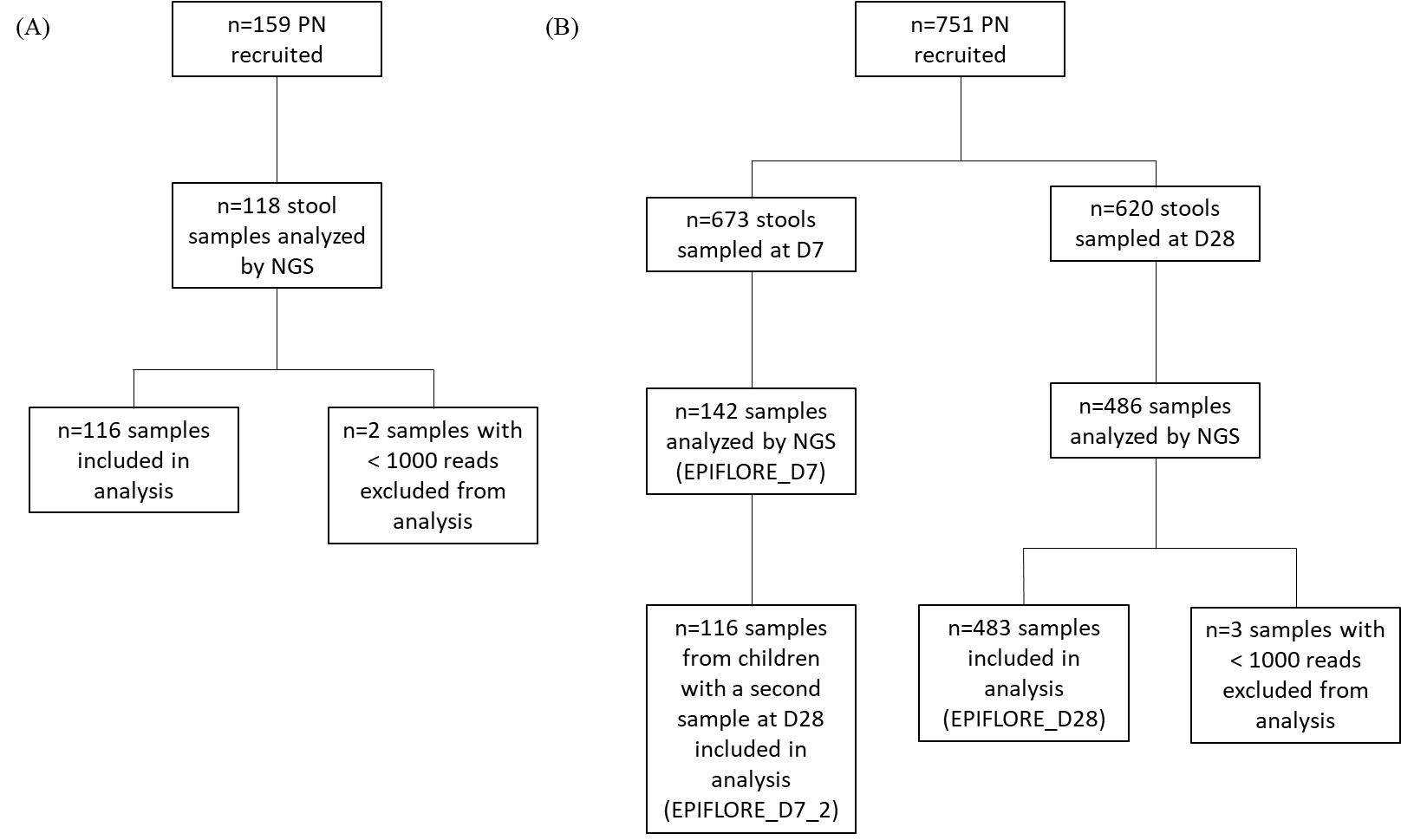
**

**Supplementary Figure S1.** Flowcharts representing the subject recruitment and stool sampling for the ClosNEC (A) and EPIFLORE (B) cohorts.


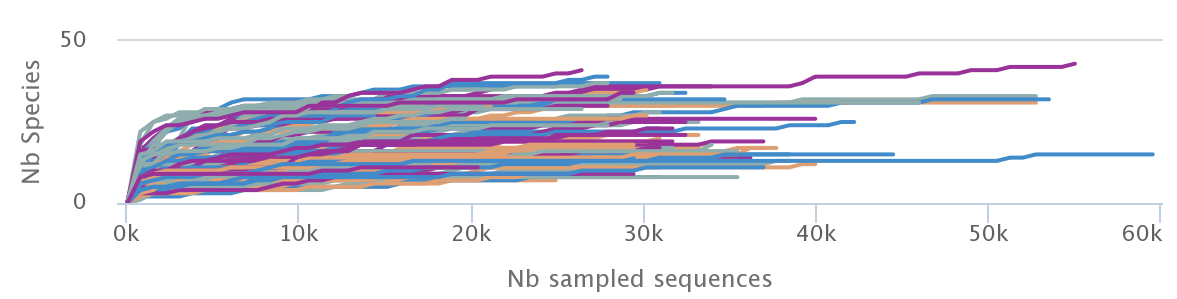


**Supplementary Figure S2.** Rarefaction curves for the ClosNEC dataset


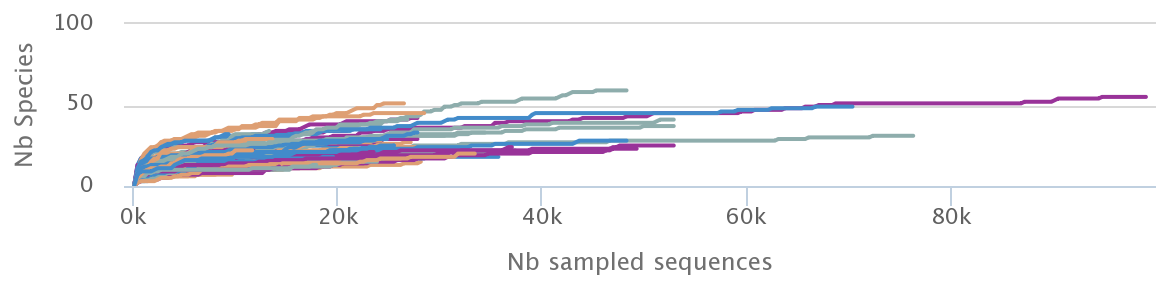


**Supplementary Figure S3.** Rarefaction curves for the EPIFLORE_D28 dataset


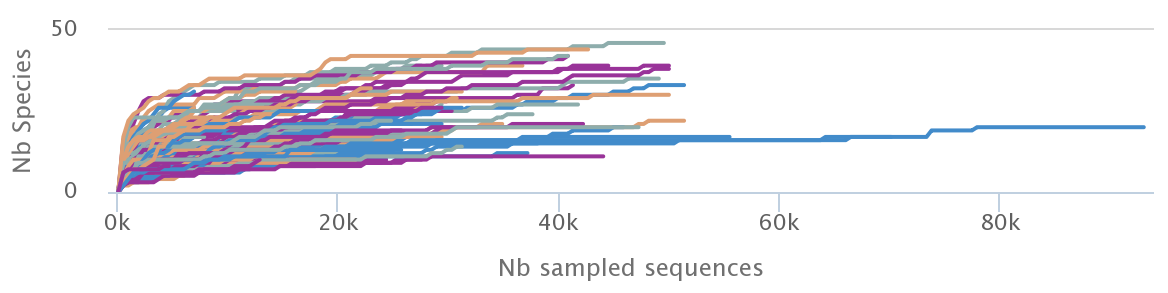


**Supplementary Figure S4.** Rarefaction curves for the EPIFLORE_D7_2 dataset


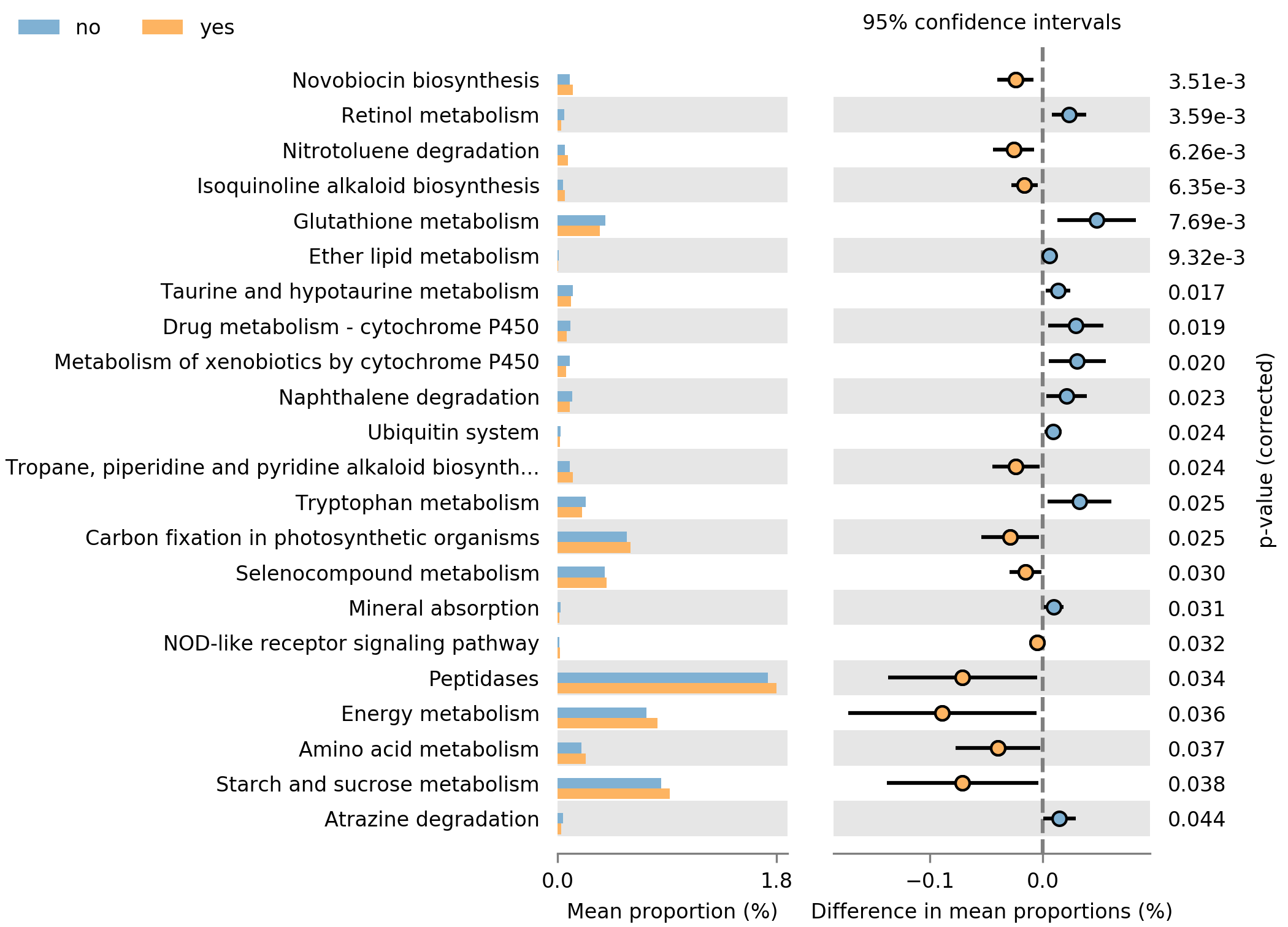


**Supplementary Figure S5.** Functionally predicted KEGG pathways differing in proportions in CD+ (yellow) and CD− (blue) groups in the ClosNEC dataset. The bar plot shows mean proportions of differential KEGG pathways predicted using PICRUSt2. The difference in proportions between the groups is shown with 95% confidence intervals. Only *p* values < 0.05 (Welch’s *t* test) are shown.

**
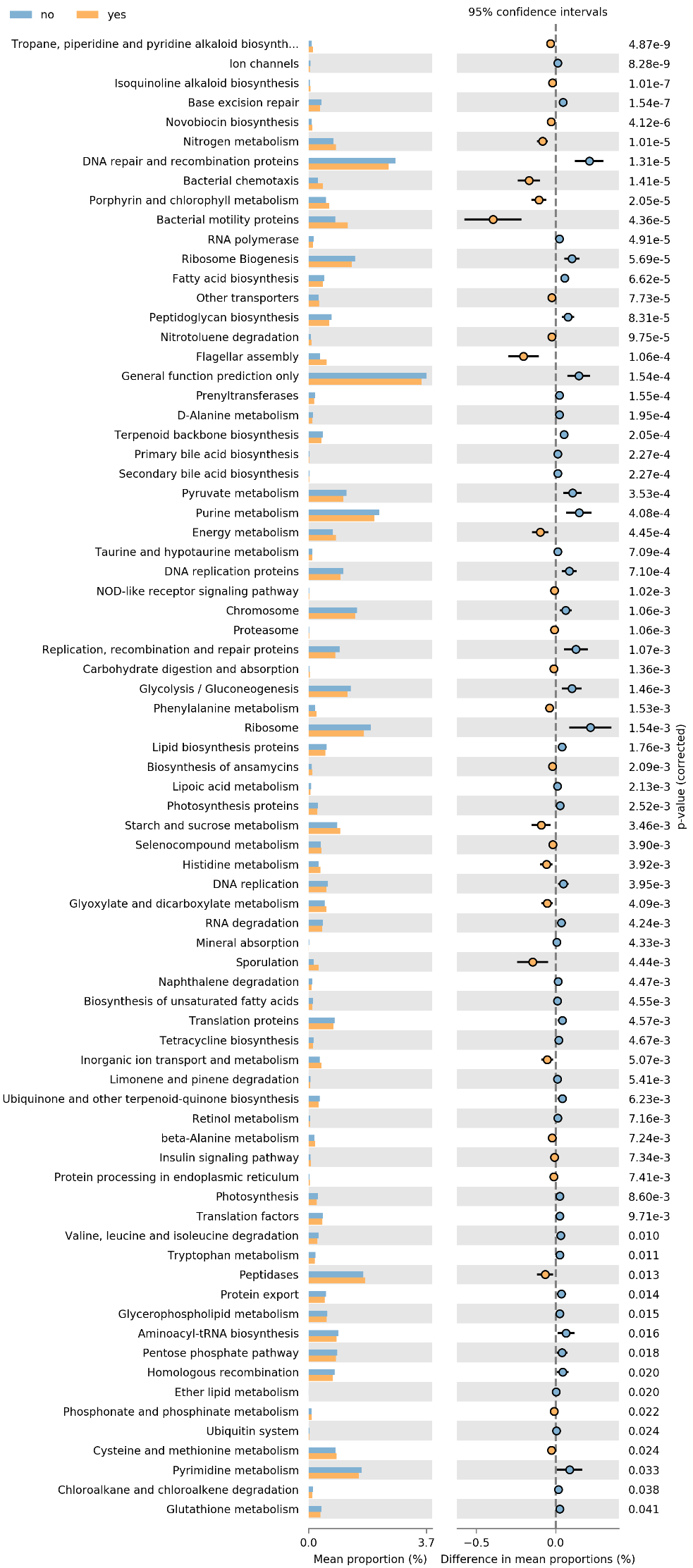
**

**Supplementary Figure S6.** Functionally predicted KEGG pathways differing in proportions in CD+ (yellow) and CD− (blue) groups in the EPIFLORE_D28 dataset. The bar plot shows mean proportions of differential KEGG pathways predicted using PICRUSt2. The difference in proportions between the groups is shown with 95% confidence intervals. Only *p* values < 0.05 (Welch’s *t* test) are shown.
