# Supplementary material for: Gut Microbiota Diversity of Preterm Neonates Is Associated With Clostridioides Difficile Colonization
Source: Front Cell Infect Microbiol. 2022 Jul 6;12:907323. doi: 10.3389/fcimb.2022.907323 (PMC9296818; doi:10.3389/fcimb.2022.907323)
Supplement: Supplementary file 2 [file Table_1.docx]

**Table S1.** Comparison of culture and NGS of the bacterial 16S rRNA gene for the detection of *C. difficile* in stools in the ClosNEC cohort.

|  | | **Culture** | |
| --- | --- | --- | --- |
|  | | N Positive (%) | N Negative (%) |
| **NGS** | N Positive (%) | 23 (19.8) | 11 (9.5) |
|  | N Negative (%) | 5 (4.3) | 77 (66.4) |

**Table S2.** Linear mixed model**:** interaction between the most frequent OTUs (*p* < 0.05) and analyzed variables. The reference group for the comparison is highlighted in bold. The most representative OTUs associated with each variable included in the model are shown, after adjusting for all factors, the last column shows the *p-*value and in parentheses the interaction association (values > 0 indicate a positive association, values < 0 indicate a negative association).

| **Cohort** | **Groups** | **n OTUs associated** | **Top OTUs associated** | ***p*** | **Adjusted *p* (interaction association)** |
| --- | --- | --- | --- | --- | --- |
| **ClosNEC** | **CD**+ / CD− | 94 | *Rothia* | 0.012 | **0.016** (0.97) |
|  |  |  | *Bifidobacterium* | 0.029 | **0.004** (2.09) |
|  |  |  | *Anaerococcus* | 0.003 | 0.052 (0.38) |
|  |  |  | *Veillonella* | 0.047 | **0.036** (0.98) |
|  |  |  | *Clostridioides* | <0.0001 | **<0.0001** (8.43) |
| **EPIFLORE_D28** | **CD**+ / CD− | 151 | Pasteurellaceae | 0.001 | 0.12 (0.39) |
|  |  |  | Enterobacterales | 0.002 | **<0.0001** (0.62) |
|  |  |  | *Eisenbergiella* | 0.003 | **0.035** (0.20) |
|  |  |  | *Morganella* | 0.004 | 0.10 (0.35) |
|  |  |  | *Sutterella* | 0.004 | 0.18 (0.13) |
|  |  |  | *Clostridioides* | <0.0001 | **<0.0001** (5.99) |
